# A case of necrotic pneumonia caused by *Streptococcus pneumoniae* was diagnosed using a pneumonia antigen test in BALF: A case report

**DOI:** 10.1097/MD.0000000000039571

**Published:** 2024-09-06

**Authors:** Yanjing Huang, Hongkun Guo, Yiming Li

**Affiliations:** a Department of Emergency, the First Affiliated Hospital, Fujian Medical University, Fuzhou, China; b Department of Emergency, National Regional Medical Center, Binhai Campus of the First Affiliated Hospital, Fujian Medical University, Fuzhou, China.

**Keywords:** lung abscess, *S. pneumoniae*, *S. pneumoniae* urinary antigen

## Abstract

**Rationale:**

*Streptococcus pneumoniae* is a common cause of community-acquired pneumonia. Currently, it is believed that many cases of pulmonary infection with negative results on pathogenic testing are caused by *S. pneumoniae*. There have been no reports of the detection of *S. pneumoniae* antigen in lung lavage fluid.

**Patient concerns:**

An elderly male patient with suboptimal fasting blood glucose control and a history of liver abscess.

**Diagnosis:**

Chest computed tomography (CT) revealed inflammatory lesions in both lungs with consolidation in the middle lobe of the right lung.

**Interventions:**

After admission, we collected alveolar lavage fluid in a timely manner and performed pneumococcal antigen detection and etiological testing.

**Outcomes:**

Prompt testing for *pneumococcal* antigen in bronchoalveolar lavage fluid yielded a positive clinical outcome. Subsequent analysis via bacterial culture of sputum and next-generation sequencing (mNGS) of BALF definitively identified *S. pneumoniae* as the etiological agent. Following the analysis of drug sensitivity test results from the identified pathogens, adjustments were made to the antibiotic regimen, and appropriate pus puncture drainage was performed. Subsequently, the patient’s condition improved, leading to discharge.

**Conclusion:**

The identification of *S. pneumoniae* antigen in bronchoalveolar lavage fluid may facilitate earlier and more precise diagnosis of pneumonia attributed to *S. pneumoniae*.

## 1. Introduction

*Streptococcus pneumoniae* infection is a common cause of community-acquired pneumonia (CAP).^[1]^ However, in most cases, pathogen detection results are negative. Pulmonary abscess is typically viewed as an infrequent complication of pneumococcal infections. While *S. pneumoniae*-associated pulmonary abscesses are prevalent in children, they are comparatively uncommon in adults. Serotype 3 *S. pneumoniae* has been identified as the primary etiological agent of necrotizing lung disease, although instances involving other serotypes have been documented.^[2,3]^

## 2. Case report

**Figure 1.**
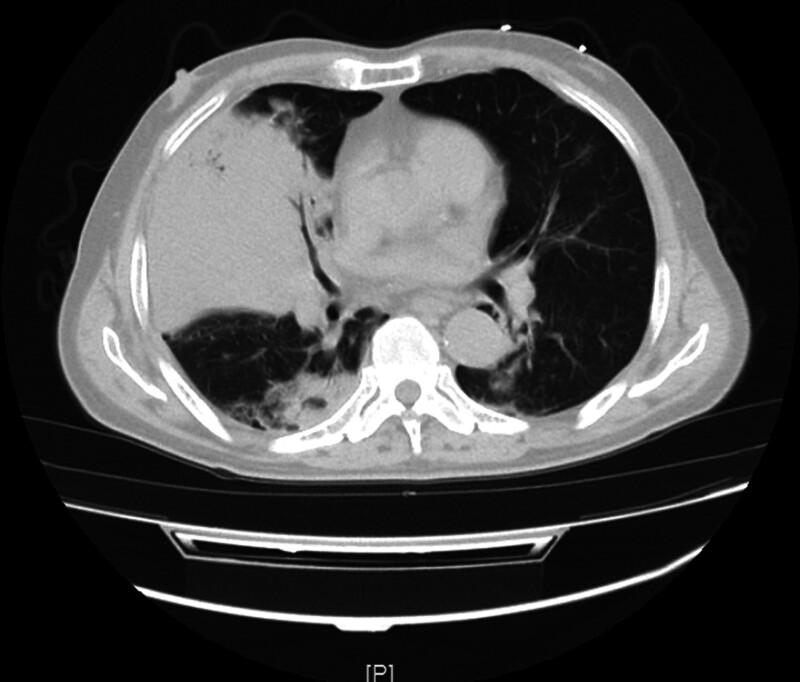
CT demonstrated inflammatory lesions in both lungs and consolidation of the right middle lobe.

A 60-year-old man presented to our hospital on March 7, 2021, with a chief complaint of 4-day right chest pain, shortness of breath, fatigue, and 1-day aggravation. Four days before admission, the patient had right chest pain after catching cold, which radiated to the right upper abdomen, accompanied by shortness of breath, occasional cough, and sputum expectoration. The blood temperature was unknown. No manifestations such as fear of cold or shivering were reported. One day before admission, the patient’s symptoms had worsened Emergency computed tomography (CT) revealed inflammatory lesions in both lungs and consolidation of the right middle lobe (RML) (Fig. 1). Laboratory examination showed a procalcitonin (PCT) of 33.11 ng/ml, C-reactive protein (CRP) level of > 90 mg/L, creatinine (Cr) of 194.3 µmol/L, white blood cell of 3.92 × 109/L, hemoglobin (Hb) level of 143 g/L, and platelet count (PLT) of 145 × 109/L. “Pulmonary infection and type I respiratory failure” was initially diagnosed. Sputum specimens were collected for bacterial culture. The patient was connected to a noninvasive ventilator for assisted breathing and administered Ertapenem + Azithromycin for anti-infection treatment. The symptoms improved. During treatment in the emergency department, the patient presented with a reoccurrence of fever, reaching a maximum temperature of 37.8 °C. The patient had a history of type 2 diabetes for 4 years and reported irregular use of metformin, gliclazide, and acarbose for blood sugar control. Despite this medication regimen, blood sugar levels remained poorly controlled, with postprandial levels ranging from 10 to 21.2 mmol/l. Two years prior, the patient had been hospitalized in the Department of Hepatology for a bacterial liver abscess and intraocular inflammation, and was discharged following improvement. Additionally, the patient reported consuming at least 200 ml liquor of heavy alcohol daily (the specific data are unknown). while there was no history of long-term smoking.

**Figure 2.**
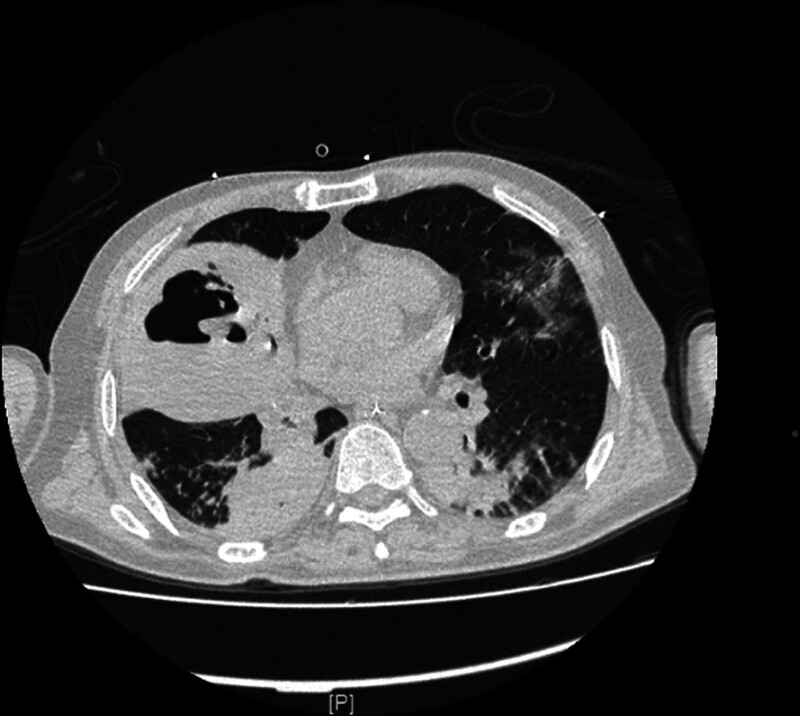
Chest CT revealed consolidation of the right middle and lower lobes (cavities with a gas–liquid interface inside were found within the lesion in the middle lobe).

Upon admission to the Emergency Intensive Care Unit (EICU) that evening, the patient exhibited exacerbation of asthma symptoms, necessitating tracheal intubation and mechanical ventilation. We placed nasogastric tubes, deep venous catheters, and urinary catheters in the patient and collected blood, sputum, and urine samples for etiological testing. Bronchoscopy and lung lavage were performed at the bedside, and the alveolar lavage fluid was collected for etiological testing. At the same time, we also conducted a *pneumococcal* antigen assay of the alveolar lavage fluid. At the same time, we also conducted a *pneumococcal* antigen test on the bronchoalveolar lavage fluid, which showed positive results. Subsequent laboratory tests after admission indicated that the pathogen was *pneumococcal*; *Adenovirus, influenza A and B viruses, respiratory syncytial virus*, and *Mycoplasma pneumoniae* were negative. The TORCH examination was negative for *Toxoplasma gondii, giant cells, herpes simplex virus,* and *rubella virus* IgM. BALF smear analysis revealed the presence of gram-positive cocci (G+), while the BALF culture exhibited bacterial autolysis without any growth. Bronchoalveolar lavage fluid smear: G + cocci. Bacterial culture of bronchoalveolar lavage fluid showed no bacterial growth, and the bacteria showed an autolytic phenomenon. A sputum bacterial culture showed *pneumococcal* + + biofilm formation. Bronchoalveolar lavage fluid was sent for mNGS(Metagenomic next-generation sequencing), and PDC-seqTM detected microorganisms, such as *pneumococci*, Detailed results: It belongs to the G + *Streptococcus* genus, with a sequence number of 925 and a relative abundance of 97.57%; species – *pneumococcal,* with a sequence number of 669 and a relative abundance of 70.57%. RNA virus rhinovirus with a relative abundance of 1.86% (Fig. 2).

**Figure 3.**
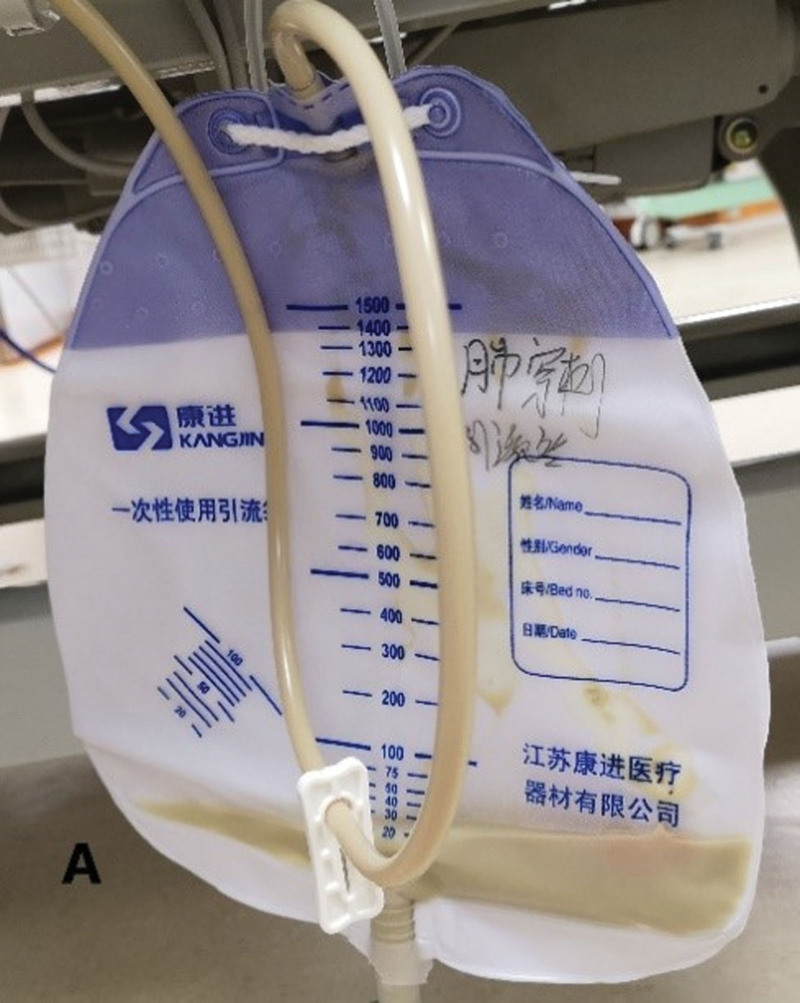
CT-guided puncture for drainage of the lung abscess.

**Figure 4.**
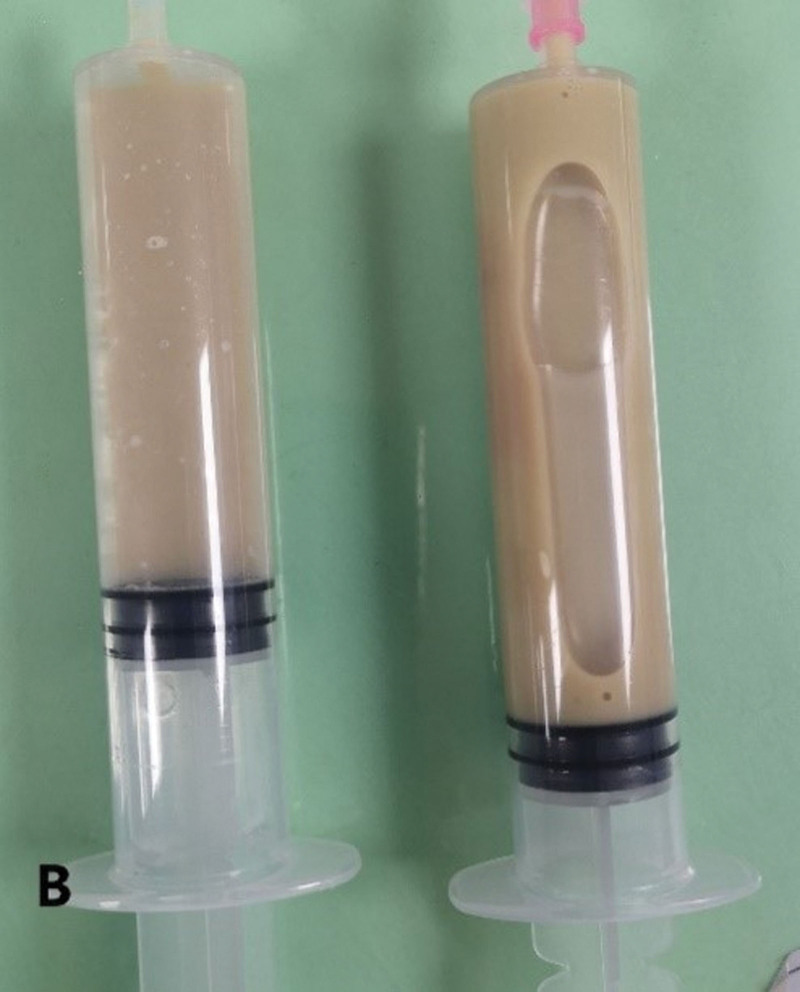
Purulent fluid extracted from chest lesion puncture.

**Figure 5.**
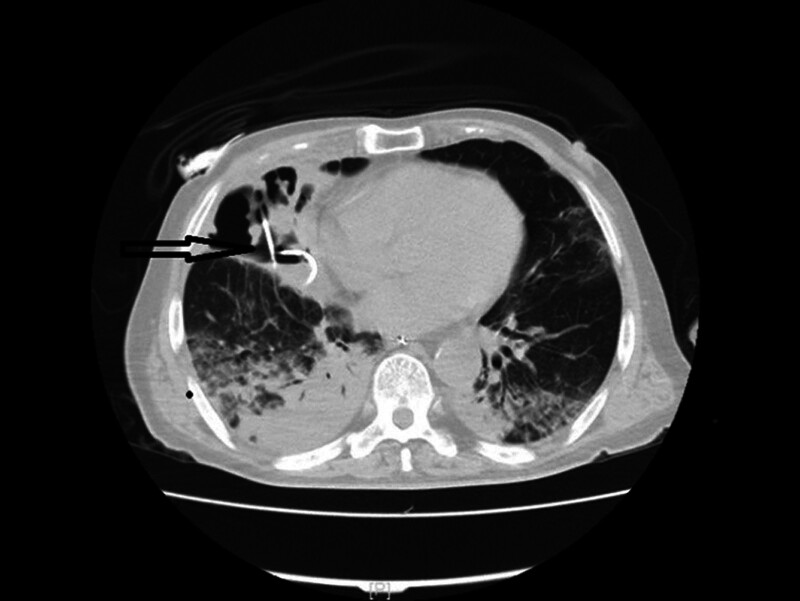
A drainage tube placed in the lesion of the right lung (arrow).

**Figure 6.**
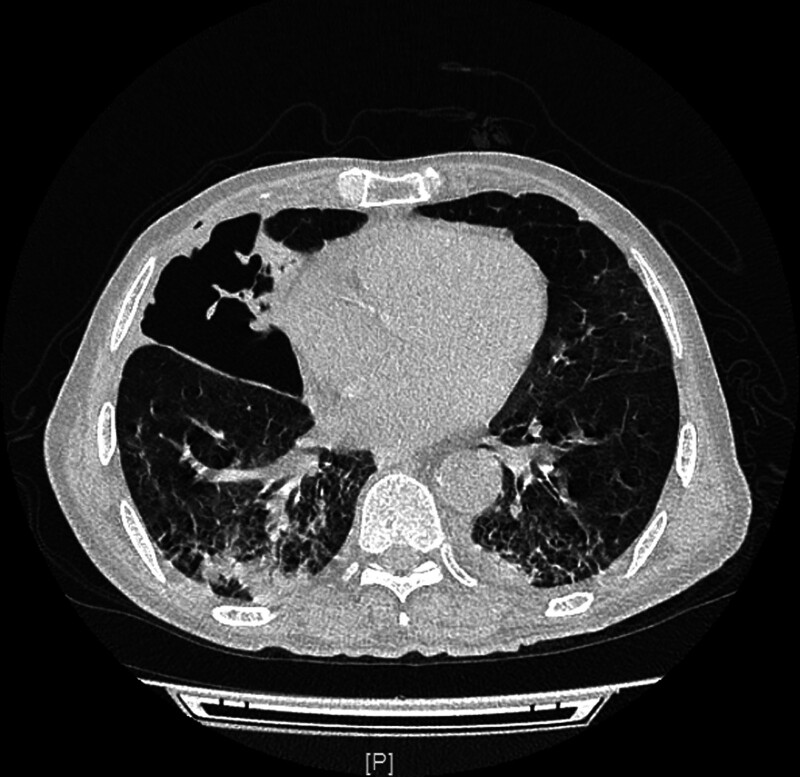
The abscess fluid was completely cleared, and the lesion was significantly absorbed after treatment.

Based on the *pneumococcal* antigen results in the bronchoalveolar lavage fluid, we promptly adjusted the antibiotic regimen from ertapenem 1.0 g qd + doxycycline 100 mg bid at admission to moxifloxacin (with the effect of breaking bacterial biofilm) 400 mg qd for anti-inflammatory treatment. After the above treatment, the patient’s condition gradually improved (Fig. 3), and the tracheal tube was removed on March 20th. On March 23rd, a CT scan of the lung showed right lung abscess formation (Fig. 4), and then a CT-guided lung abscess puncture and drainage procedure was performed (Figs. 5 and 6). After treatment, the patient’s condition improved, and after reviewing the lung CT on April 30th, the patient recovered and was discharged.

## 3. Discussion

Lung abscess is a pathological condition characterized by chronic necrotic alterations in the lung parenchyma, resulting from localized necrosis and suppuration caused by infection with various pathogens, primarily bacteria. This condition is also referred to as necrotizing pneumonia or lung gangrene.^[4]^ Additionally, pulmonary infection leads to inflammation and formation of dense consolidations within the lungs. The presence of cavitary lesions in lung parenchyma is a distinctive feature of lung abscess.^[5,6]^ Upon formation of a lung abscess, inadequate tissue perfusion over a large area results in poor antibiotic delivery to the lesion site, potentially leading to tissue liquefaction and gangrene formation. Therefore, in most cases, surgery is required. *S. pneumoniae, Staphylococcus aureus*, and *Klebsiella pneumoniae* are the most common pathogens that cause lung abscesses. While *S. pneumoniae* is a prevalent bacterium responsible for CAP, the occurrence of lung abscess formation by this pathogen is infrequent.^[7]^

The extent of *S. pneumoniae*-induced diseases is associated with bacterial virulence and host factors. Since *S. pneumoniae* is unable to produce necrotizing toxins, the mechanism by which it causes lung necrosis remains elusive.^[8]^ Wang et al^[9]^ believed that hematogenous dissemination causes bacteria to proliferate within the pulmonary vascular walls, resulting in arterial and venous thromboembolism, and eventually, lung necrosis. In a retrospective study by Maitre et al,^[10]^ 68 patients with lung abscess (male: n = 47, 73.4%) were included, and the right lower lobe (RLL) of the lung was found to be the most commonly affected site in 33% of the patients. In addition, a large proportion of patients had alcoholism (40%) and a risk of inhalation of other factors (14%), which led to unconscious intake of oral secretions, especially by the RLL (as the right bronchus is straighter than the left bronchus in anatomy). A study by Nuri et al^[11]^ compared 2 cohorts of patients with community-acquired lung abscess (CALA) (n = 44) and nosocomial lung abscess (NLA) (NLA; n = 18). The lesion location included the right upper lobe (RUL) in 31.8% and RLL in 29.5% of cases in the CALA group, while the lesions in the NLA group were mostly located in the RUL, left upper lobe, and left lower lobe in of 27.8%. *S. aureus* (n = 4, 20.0%) was the most common microorganism in the CALA group, followed by *S. pneumoniae* (n = 3, 15.0%) and *Pseudomonas aeruginosa* (n = 3, 15.0%). In our patient, consolidation of the the RML was initially observed (Fig. 1), followed by lung necrosis. Chest CT showed cavitation in the lesion of the RML, within which a gas–liquid interface was observed (Fig. 2). Risk factors for invasive *pneumococcal* disease (IPD) include age < 2 or > 65 years, presence of comorbidities (e.g., DM, renal insufficiency, or nephrotic syndrome), excessive drinking, and immunosuppression.^[12]^ The patient was a 60-year-old man with poor glycemic control of fasting blood glucose and a history of DM, long-term alcohol abuse (at least 200 mL liquor daily), and hepatic bacterial abscess, all of which are risk factors for necrotizing pneumonia in this patient.

*S. pneumoniae* is a Gram-positive, type A hemolytic bacterium that is difficult to cultivate and grows best in a 5% carbon dioxide environment. During the normal growth period in broth, when the cultured bacteria reach a high density, an autolytic enzyme is triggered, causing characteristic autolysis and death of all bacteria in the culture medium. A rapid test for the detection of urinary antigens has been proven to facilitate the diagnosis of *S. pneumoniae*-associated CAP.^[13]^ However, a recent study showed that *S. pneumoniae* was detected in only 5% to 15% of pneumonia cases in the United States. There may be 2 reasons for this result: sputum specimen from 15% to 30% of patients were not available andantibiotics were used in approximately 25% of patients before specimen collection.^[14,15]^ Perazzo et al^[16]^ documented 2 instances of lung abscesses attributed to *S. pneumoniae*. Despite initial negative results from blood culture and urine antigen testing for *S. pneumoniae* upon admission, the etiology of the lung abscesses was ultimately confirmed as *S. pneumoniae* through analysis of sputum obtained via bronchoscopy.

Nicolini et al,^[17]^ 3 cases of lung abscesses attributed to *S. pneumoniae*. Among these cases, 2 patients initially tested negative for *S. pneumoniae* in blood bacterial culture and urine antigen detection following admission; however bronchoscopy-aided pathogenic analysis of sputum revealed the presence of *S. pneumoniae.* Conversely, 1 patient exhibited a positive result in urine antigen detection for *S. pneumoniae* promptly after admission, and timely identification of the causative microorganisms is critical for the selection of suitable antibiotics, providing sufficient time for administration, and affecting patient prognosis.^[18,19]^ In this case, in order to promptly ascertain the etiology of the disease, sputum samples were obtained using a fiberoptic bronchoscope upon admission and at the patient’s bedside, followed by subsequent etiological analysis.

Although bacterial culture of the bronchoalveolar lavage fluid showed no bacterial growth and the bacteria appeared to be autolytic, the *pneumococcal* antigen test yielded a positive result (Fig. 2). This finding was further supported by subsequent sputum bacterial culture and metagenomic next-generation sequencing (mNGS) testing, which confirmed the presence of *S. pneumoniae* as the causative pathogen. After reviewing the available literature, we did not identify any studies reporting the detection of *S. pneumoniae* antigen in alveolar lavage fluid. But Devresse A, Seront B, et al demonstrated rapid diagnosis of *pneumococcal* infection through the detection of *pneumococcal* antigen in peritoneal fluid.^[20]^ Zhang et al utilized cerebrospinal fluid samples to detect pneumococcal antigen, resulting in positive diagnoses of pneumococcal infection.^[21]^ Aston et al reported positive antigen testing for *S. pneumoniae* in pleural fluid samples from patients, confirming the presence of pneumococcal infection.^[22]^ Therefore, we believe that the identification of *pneumococcal* antigens in bronchoalveolar lavage fluid could potentially enhance the timeliness and precision of *pneumococcal* pneumonia diagnosis. Further studies are required to substantiate this claim.

## Author contributions

**Data curation:** Hongkun Guo.

**Writing—original draft:** Yanjing Huang, Hongkun Guo.

**Writing – review & editing:** Yiming Li.
